# The effect of iron dextran on vitamin D_3_ metabolism in SD rats

**DOI:** 10.1186/s12986-022-00681-5

**Published:** 2022-07-16

**Authors:** Fubin Qiu, Rui Li, Siyu Gu, Yimin Zhao, Linxue Yang

**Affiliations:** Department of Nutrition and Food Hygiene, School of Public Health, Shanxi Medical University, 56 Xinjian South Road, Taiyuan, China

**Keywords:** Iron, 25-(OH)D_3_, 1,25-(OH)_2_D_3_, CYP2R1, CYP27A1, CYP27B1, CYP24A1

## Abstract

**Background:**

Iron and vitamin D (VD) is essential to health. Previous studies have shown that iron homeostasis has a potential effect on VD metabolism, but the mechanism is not fully understood.

**Objectives:**

To explore the relationship between VD metabolism and iron metabolism, as well as the regulatory mechanism of iron on VD metabolism.

**Methods:**

40 male rats were fed adaptively for 7 days and randomly divided into control (C, n = 6 normal diet) group and model (M, n = 24 iron deficient diet) by simple randomization, the latter was used to establish iron deficiency anemia (IDA) model. After 6 weeks of feeding, the M group was randomly divided into: iron deficiency group (DFe), low iron group (LFe), medium iron group (MFe) and high iron group (HFe) by block randomization. Different doses of iron dextran (based on iron content (100 g·bw·d)): 0, 1.1, 3.3 and 9.9 mg) were given respectively. After 4 weeks, the rats were anesthetized with 8% chloral hydrate, Blood (collected from the abdominal aorta), liver and kidney tissues were collected. The serum and tissues were separately packed and frozen at -80℃ for testing.

**Results:**

The results showed that the levels of hemoglobin (Hb), red blood cell (RBC), serum iron (SI), liver iron, and kidney iron in DFe group were lower than those in the other four groups, while the levels of total iron-binding capacity (TIBC), transferrin (TF) and transferrin receptor (Tfr) in DFe group were higher than those in other groups; The serum levels of 25-(OH)D_3_ and 1,25-(OH)_2_D_3_ in DFe group were significantly lower than those in C group (*P* < 0.05). The correlation analysis showed that the levels of 25-(OH)D_3_ and 1,25-(OH)_2_D_3_ were negatively correlated with TIBC, TF and Tfr no correlation with SI. Western blotting, immunofluorescence, and q-PCR results showed that compared with C group, the protein and gene expressions of CYP2R1, CYP27A1, and CYP24A1 in DFe group were down-regulated, and the expression of CYP27B1 protein and gene was up-regulated in DFe group.

**Conclusion:**

Iron may be involved in the metabolism of VD_3_ by regulating the expression of VD_3_ hydroxylase, suggesting that appropriate iron supplementation might promote the activation of VD_3_.

**Supplementary Information:**

The online version contains supplementary material available at 10.1186/s12986-022-00681-5.

## Introduction

Vitamin D (VD) is an essential micronutrient for the body, which is vital to health. In addition to regulating calcium and phosphorus metabolism, VD with hormone properties can induce cell differentiation and apoptosis, inhibit cell proliferation and other cell signal transduction processes, participate in the regulation process of multiple genes, and involve in the physiological and pathological mechanism of various diseases [1, 2]. VD deficiency is related to many diseases, including immune dysfunction, metabolic syndrome, insulin resistance, infection, cancer, and cardiovascular abnormalities [3–5]. According to statistics, about 30% and 60% of children and adults in the world are deficient in VD [6]. There is also a widespread VD deficiency in Chinese population. Ling et al. [7] surveyed elderly people over 60 years old in different latitudes in our country, and showed that 92.28% of the elderly suffer from VD deficiency and insufficient; Xu's [8] findings suggest that 62.40% of young males and 52.39% of young females in East China had VD deficiency. VD is generally lacking in people all over the world, even in high sunshine areas such as Algeria, Libya, and Brazil [9–11].

VD is a fat-soluble vitamin, including five types of VD_1_-VD_5_, among which VD_3_ (cholecalciferol) and VD_2_ (ergosterol) have the greatest impact on human health [12]. Endogenous synthesis of VD_3_ through ultraviolet rays exposure to cholesterol precursors in the skin. The liver 25-hydroxylase (CYP2R1 and CYP27A1 enzymes) catalyzes the formation of 25-(OH)D_3_, and the circulating 25-(OH)D_3_ is further hydroxylated in the kidney, depending on 1α-hydroxylase (CYP27B1 enzyme). The active form of VD (1,25-(OH)_2_D_3_) combines with VDR to play a biological role in the body, and CYP24A1 participates in the catabolism of VD [13]. The activated VD can promote cell differentiation and proliferation, bone resorption, and improve immunity [14]. The search for continuous and stable 25-(OH)D_3_ and highly active 1,25-(OH)_2_D_3_ has always been the focus of research.

Iron is another essential micronutrient and a cofactor of many enzymes, which participates in many physiological processes, especially in hemoglobin production, heme synthesis, electron transfer, and so on. More and more observational studies have shown that there is an association between VD deficiency and iron deficiency or iron load. The third national health and nutrition survey found that VD deficiency was related to anemia [15]. Atkinson et al. [16] pointed out that 25-(OH)D_3_ deficiency was associated with an increased risk of anemia. Katsumata et al. [17] have shown that iron deficiency leads to VD deficiency, and iron plays an important role in VD synthesis. In iron overload diseases, VD deficiency is related to iron accumulation in the liver [18]. Based on these findings, iron homeostasis may affect the metabolic level of VD. But so far, the exact mechanism of the regulation of iron on VD metabolism has not been reported. Therefore, it is of great significance to explore the regulatory mechanism of iron on VD metabolism for VD deficiency caused by iron homeostasis imbalance.

In our study, iron deficiency anemia (IDA) rat model was used to determine the relationship between VD and iron metabolism; we hypothesized that iron deficiency would down-regulated the metabolism level of VD, and iron loading would inhibit the metabolism level of VD; iron might affect the metabolism level of VD through the regulation of hydroxylase.

## Methods

### Experimental designs

The 40 male newly weaned Specific Pathogen Free (SPF) SD rats, weighing 66.46 ± 4.22 g, were purchased from Beijing Changyang Xishan animal farm, animal Certificate No: scxk (Beijing): 2019-0010. After 1 week of adaptive feeding, they were randomly divided into control (C, n = 8) group and model (M, n = 32) group by simple randomization. C group was fed with normal diet and normal drinking water; M group was fed with iron deficiency diet (iron content was 21.33 mg/kg) and drinking deionized water to establish IDA model. After 6 weeks, they were randomly divided into four groups: iron deficiency group (DFe) [0 mg/(100 g·bw·d)], low iron group (LFe) [1.1 mg/ (100 g·bw·d)], medium iron group (MFe) [3.3 mg/(100 g·bw·d)], high iron group (HFe) [9.9 mg/(100 g·bw·d)] by block randomization, 2 ml for each animal for 4 weeks, C and DFe groups were perfused with 2 ml normal saline. In addition to the different iron content, the other nutritional components of the two diets were the same. Both of them added 1500 IU of VD_3_, and used shading cloth to prevent the rats from sunlight to synthesize VD_3_ through the skin. The temperature and relative humidity were 21 ± 1℃ and 46–60% respectively. In order to avoid iron pollution, SD rats were housed in stainless steel cages, with stainless steel food containers and plastic drinking bottles. After 4 weeks of intervention, the rats were anesthetized with 8% chloral hydrate. Blood samples were collected from the abdominal aorta for 8–10 ml. The blood was placed in a centrifugal tube for 30 min at room temperature and then centrifuged at 3000 r/min for 10 min to separate the serum. After washing the liver and kidney with normal saline, some of them were fixed by immersion in 4% paraformaldehyde, and the others were put into nuclease free tube and frozen at −80℃.

### Main reagents and instruments

Iron Dextran (Yuanye Biology, S51662); VD_3_ (Beijing Solarbio, V8070); Tissue Iron Determination Kit (Nanjing Jiancheng, A039-2-1); hemoglobin (Hb) Kit (Nanjing Jiancheng, C021-1-1); red blood cell (RBC) diluent (Leagene Biotechnology, DA0150); 25-(OH)D_3_ kit (Jianglai Biological, JL20734); 1,25-(OH)D_3_ kit (Jianglai Biological, JL27246); transferrin (TF) (Jianglai Biotechnology, JL-31079); transferrin receptor (Trf) (Jianglai Biotechnology, JL-30566); serum iron (SI) (Nanjing Jiancheng, A039-1-1); iron-binding capacity (TIBC) (Nanjing Jiancheng, A040-1-1); CYP2R1 primary antibody (ABclonal, A10470); CYP27A1 primary antibody (ABclonal, A1982); CYP27B1 primary antibody (ABclonal, A1716); CYP24A1 primary antibody (ABclonal, A1805); Cy3-goat anti-rabbit IgG (Beyotime, A0516). Microplate reader (Bio-Rad, USA); DAPI (Aladdin, D106471); Paraffin embedding machine (Wuhan Junjie Electronics Co, Ltd, JT-12S); Microtome (Lycra Microsystems Co, Ltd, RM2016); Electron microscope (Nikon Instruments Co, Ltd, Eclipse Ci-L); PCR instrument (Hangzhou Bioet Company, Line Gene9640PCR).

### Detection of hematological and serological indexes

ELISA kit was used to detect 25-(OH)D_3_, 1,25-(OH)_2_D_3_, TF, and Tfr; Biochemical kit was used to detect Hb, RBC,TIBC, SI, liver iron and kidney iron, and the operation was carried out strictly according to the operation procedures of the kit.

### Western blotting was used to detect the expressions of CYP2R1, CYP27A1, CYP27B1 and CYP24A1 protein

The expressions of CYP2R1 and CYP27A1 protein in liver, and the expressions of CYP27B1 and CYP24A1 protein in kidney were detected, each sample was taken 20 mg, and Radio Immunoprecipitation Assay (RIPA) lysate was added, The total protein was extracted, protein was prescribed by BCA Assay Kit, the total protein was sampled with 20 μg protein in 96-well plate, gel electrophoresis, transfer membrane, block, 4℃ primary antibody CYP2R1, CYP27A1, CYP27B1, CYP24A1 (1:500), β-actin (1:5000) were incubated overnight at room temperature, The secondary antibody (1:5000) was incubated for 2 h, CYP2R1, CYP27A1, CYP27B1, and CYP24A1 were detected by the luminescent solution, and the gray value was analyzed by Image J software.

### Immunofluorescence staining of CYP2R1, CYP27A1, CYP27B1 and CYP24A1 protein

The liver and kidney tissue specimens fixed with 4% paraformaldehyde, and then dehydrate after fixation, wax embedding, sectioning, dewaxing, hydration, gradient ethanol elution, antigen retrieval, CYP2R1, CYP27A1, CYP27B1, and CYP24A1 (diluted 1:100) primary antibody incubation, incubated with secondary antibody (diluted at 1:200) and counter-stain with diamidino-2-pheny1 indole (DAPI). The immunofluorescence intensity in the liver tissue was observed and photographed under a fluorescence microscope. The CYP2R1, CYP27A1, CYP27B1 and CYP24A1 protein staining were positive in red. The fluorescence intensity of CYP2R1, CYP27A1, CYP27B1 and CYP24A1 protein was detected by Image Pro Plus image analysis software, and the integrated optical density (IOD) was calculated as the final average immunofluorescence intensity.

### The mRNA expressions of CYP2R1, CYP27A1, CYP27B1 and CYP24A1 were detected by q-PCR

Total RNA was extracted from 20 mg liver and kidney tissues of rats. The cDNA was reverse transcribed and amplified by fluorescent quantitative PCR with β-actin as an internal reference. The upstream primer sequence of β-actin was 5´-TGTGATGGTGGGAATGGGTCAG-3´, downstream primer sequence 5´-TTTGATGTCACGCACGATTTCC -3´. CYP2R1 upstream primer sequence: 5´-TGCTACTACTCGTGCTGGTGGTC-3´, downstream primer sequence 5´-AGGGCCAGGGA GCAGATGTTG-3´. CYP27A1 upstream primer sequence: 5´-TCGCACCAATGTGAATCTGGC TAG-3´, downstream primer 5´-CTTCCACTGCTCCATGCTGTCTC-3´. CYP27B1 upstream primer sequence: 5´-GCACATAAACGGCAAGGCAAGTC-3´, downstream primer sequence 5´-AGCCAAGCCTCTCACCTCCTATG-3´. CYP24A1 upstream primer sequence 5´-GGAACTGT A CGCCGCTGTCAC-3´, downstream primer 5´-GCACGCTCTGAACTTCCTGAAGG-3´. PCR conditions: pre-denaturation 95℃, 30 s; denaturation: 95℃, 5 s, 60℃, 30 s (40 cycles); annealing: 95℃, 5 s, 60℃, 30 s, 95℃, 1 min; extension: 50℃, 30 s. The relative quantitative values were calculated according to the Ct values of the original q-PCR data.

### Statistical analysis

Spss 25.0 software was used for statistical analysis of the data. The HB value and body weight of group C and group M were tested by t test; VD_3_ metabolism index, iron metabolism index, tissue iron and other indicators were compared by one-way ANOVA, LSD-t test or Dunnett's T3 test were used for pairwise comparison, *P* < 0.05 means the difference was statistically significant.

## Results

### General condition of SD rats

**Fig.1:**
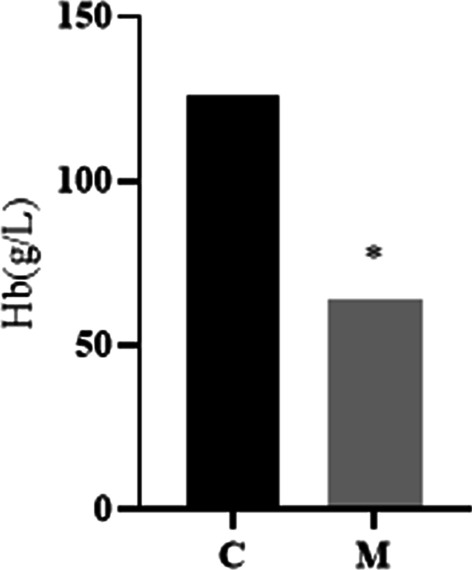
The level of Hb in C group and M group before Intervention. ^*^P < 0.05 vs C group

**Fig. 2:**
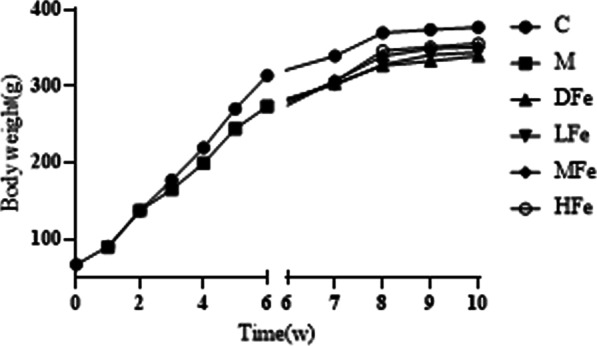
Weight changes of SD rats in each group. Adaptive feeding: 0-1w; Molding: 1-6w; Intervention: 6-10w

During the modeling period, the animals appeared some phenomena, such as fluffy hair, falling off, dull eyes, loose stool, fear of cold, white teeth, and slow growth and development and so on. After 6w of modeling, Hb in group M was detected to be lower than that in group C (63.61 ± 5.39 g/L) vs (125.58 ± 2.90 g/L) to judge the modeling situation (Fig. 1). Except for DFe group, the condition of rats in each group recovered after intervention with the corresponding dose of iron dextran. The higher the intervention concentration, the faster the weight gain (Fig. 2, Additional file 1).

### Tissue iron content

The contents of liver iron and kidney iron in DFe group were much lower than those in other four groups (*P* < 0.05), and those in LFe group were significantly lower than those in HFe group (*P* < 0.05) (Table 1).

**Table 1:** The level of tissue iron ($\overline{x}$ ± s, n = 8)

Groups	Liver iron (μmol/g)	Kidney iron (μmol/g)
C	0.60 ± 0.18	0.44 ± 0.05
DFe	0.32 ± 0.03^a^	0.33 ± 0.03^a^
LFe	0.44 ± 0.13	0.39 ± 0.06
MFe	0.66 ± 0.16 ^b^	0.44 ± 0.05 ^b^
HFe	0.77 ± 0.11^bc^	0.50 ± 0.06 ^bc^

^a^*P* < 0.05 vs C group

^b^*P* < 0.05 vs DFe group

^c^*P* < 0.05 vs LFe group

### Hematological indicators

Compared with the C group, the Hb and RBC contents were the lowest in the DFe group. The Hb content in the DFe group was significantly lower than that in the C, LFe, MFe and HFe groups, and the RBC content was significantly lower than that in the HFe group, and the differences were statistically significant (*P* < 0.05) (Table 2).

**Table 2:** Hematological indexes (μmol/g) ($\overline{x}$ ± s, n = 8)

Groups	RBC (×10^10^/L)	Hb (g/L)
C	685.00 ± 166.22	113.99 ± 12.97^*^
DFe	465.00 ± 226.21	52.04 ± 10.00
LFe	690.00 ± 406.10	106.42 ± 13.79^*^
MFe	965 ± 374.28	115.64 ± 18.72^*^
HFe	1570.00 ± 749.48^*^	121.33 ± 13.63^*^

*P < 0.05 vs DFe group

### Serum levels of 25-(OH)D_3_ and 1,25-(OH)_2_D_3_

The levels of 25-(OH)D_3_ and 1,25-(OH)_2_D_3_ in serum of DFe group were much lower than those in C, MFe and HFe groups, and the differences were statistically significant (*P* < 0.05). The content of 25-(OH)D_3_ and 1,25-(OH)_2_D_3_ gradually increased in the LFe, MFe and HFe groups, with the increase of iron intervention concentration, and the difference between the LFe group and the HFe group was statistically significant (*P* < 0.05); The content of 25-(OH)D_3_ in MFe group was significantly different from that in HFe group (*P* < 0.05) (Table 3).

**Table 3:** Serum levels of 25-(OH)D_3_ and 1,25-(OH)_2_D_3_ in SD rats ($\overline{x}$± s, n = 8)

Groups	25-(OH)D_3_(ng/mL)	1,25-(OH)_2_D_3_(ng/mL)
C	44.61 ± 8.63	29.81 ± 9.50
DFe	25.89 ± 3.96^a^	12.19 ± 6.06^a^
LFe	32.65 ± 3.87^ab^	15.47 ± 4.56^a^
MFe	35.36 ± 5.72^b^	22.98 ± 6.84^b^
HFe	49.31 ± 5.68^bcd^	29.75 ± 8.68^bc^

^a^*P* < 0.05 vs C group

^b^*P* < 0.05 vs DFe group

^c^*P* < 0.05 vs LFe group

^d^*P* < 0.05 vs MFe group

### Serum iron metabolism indicators

The levels of TIBC, TF, and Tfr were the highest in the DFe group. Compared with the C group, the levels of TIBC and Tfr were significantly increased in the DFe group, and the differences were statistically significant (*P* < 0.05); The level of SI was the lowest in the DFe group, which was significantly lower than that in the C, LFe, MFe and HFe groups (*P* < 0.05); compared with the HFe group, the level of Tfr was significantly increased in the DFe and LFe groups (*P* < 0.05) (Table 4).

**Table 4:** The serum iron metabolism indexes of SD rats ($\overline{x}$ ± s, n = 8)

Groups	TNBI (*μ*mol/L)	SI (*μ*mol/L)	TF (g/L)	Tfr (nmol/L)
C	148.72 ± 30.35	99.85 ± 16.64	5.74 ± 1.27	32.99 ± 13.83
DFe	222.28 ± 26.43^a^	36.15 ± 7.28^a^	6.73 ± 0.94	56.23 ± 14.39^a^
LFe	132.09 ± 19.24^ab^	119.48 ± 42.73^b^	6.71 ± 1.11	48.84 ± 9.07
MFe	123.45 ± 27.48^b^	93.66 ± 26.17^b^	6.03 ± 0.93	35.52 ± 12.11
HFe	86.67 ± 16.03^abc^	73.34 ± 19.75 ^b^	5.59 ± 0.89	32.7 ± 10.00^bc^

^a^*P* < 0.05 vs C group

^b^*P* < 0.05 vs DFe group

^c^*P* < 0.05 vs LFe group

### Correlation analysis of iron metabolism and VD metabolism

Correlation analysis showed that 25-(OH)D_3_ level was significantly negatively correlated with TIBC, TF and Tfr indicators; 1,25-(OH)_2_D_3_ level was significantly negatively correlated with TIBC, TF and Tfr indicators; However, 25-(OH)D_3_ and 1,25-(OH)_2_D_3_ levels had no correlation with SI (Table 5).

**Table 5:** Correlation among 25-(OH)D_3_,1,25(OH)_2_D_3_ and each variable ($\overline{x}$ ± s, n = 8)

Variables	25-(OH)D_3_	1,25-(OH)_2_D_3_
TNBI	−0.667**	−0.548**
SI	–	–
TF	−0.366*	−0.469**
Tfr	−0.491**	−0.500**

*P < 0.05

**P < 0.01

"—"No correlation

### Western blotting was used to detect the expression of CYP2R1, CYP27A1, CYP27B1, CYP24A1 protein

**Fig. 3:**
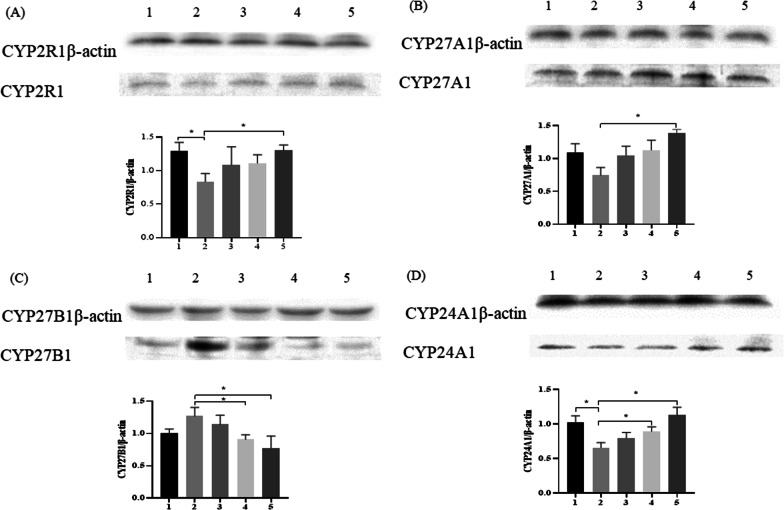
The protein expressions of CYP2R1, CYP27A1, CYP27B1, CYP24A1. n = 4, (**A**) CYP2R1, (**B**) CYP27A1, (**C**) CYP27B1, (**D**) CYP24A1; 1:C, 2:DFe, 3:LFe, 4:MFe, 5:HFe, ^*^P < 0.05

Compared with the C and HFe groups, the CYP2R1 protein expression level in the DFe group was significantly down-regulated (*P* < 0.05). With the increase of iron intervention concentration, the CYP2R1 protein expression level gradually increased (Fig. 3A); the CYP27A1 protein expression in the DFe group was the lowest in DFe group, compared with the MFe group and the HFe group, the difference was statistically significant (*P* < 0.05)(Fig. 3B); CYP27B1 was the highest in DFe group, and as the iron intervention concentration increased, the CYP27B1 protein expression level gradually decreased (Fig. 3C); compared with the C, MFe and HFe groups, the CYP24A1 expression was significantly down-regulated (*P* < 0.05)(Fig. 3D).

### Immunofluorescence staining of CYP2R1, CYP27A1, CYP27B1, CYP24A1 protein

**Fig. 4:**
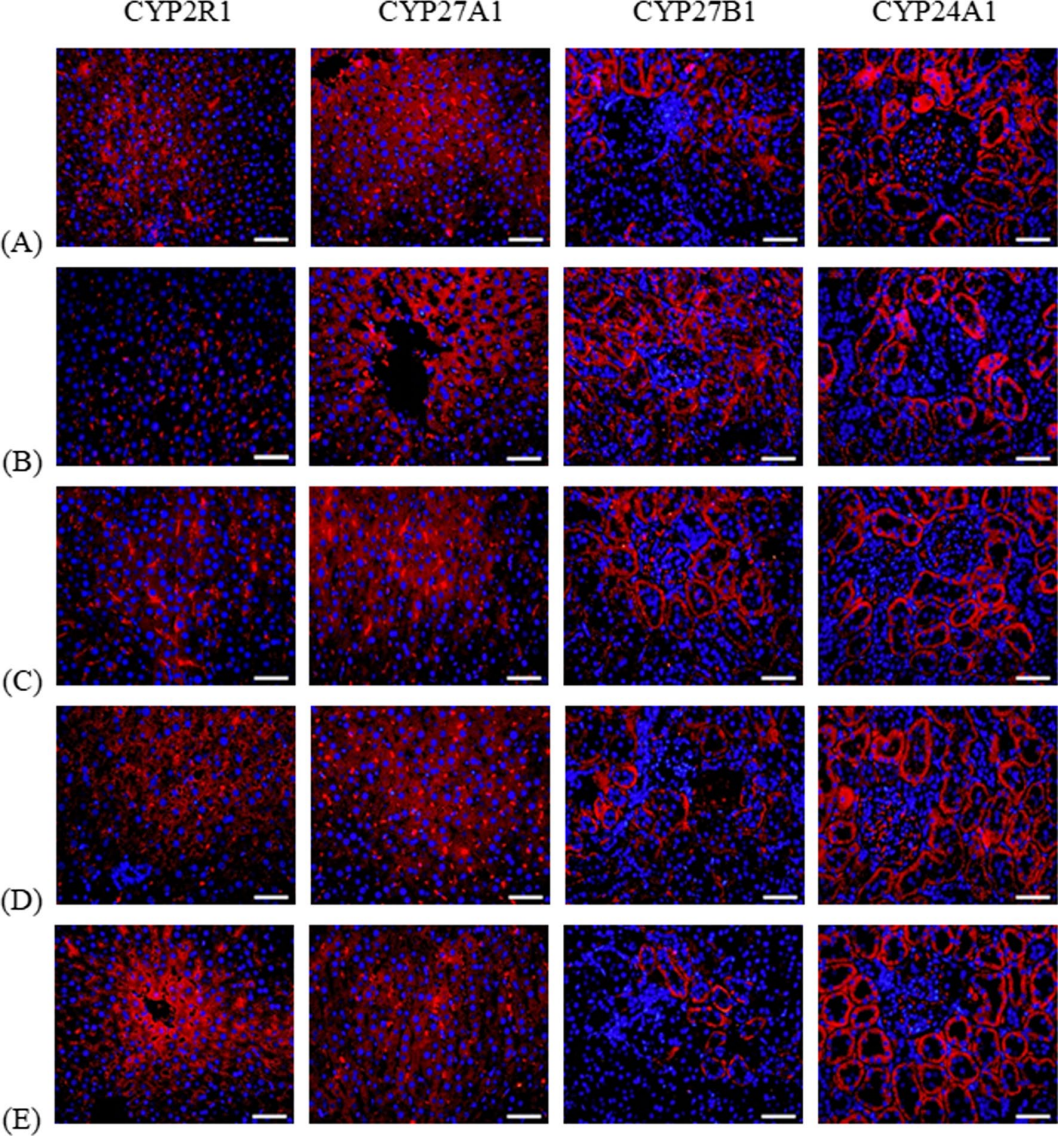
The Immunofluorescence staining of CYP2R1, CYP27A1, CYP27B1, CYP24A1(400X). n = 3, (**A**) C, (**B**) DFe, (**C**) LFe, (**D**) MFe, (**E**) HFe, Scale:50 μm

The immunofluorescence results showed that compared with C group, CYP2R1 protein expression in DFe group was significantly lower (*P* < 0.05); CYP2R1 protein expression in DFe group was significantly lower than that of MFe and HFe groups (*P* < 0.05). Compared with C group, CYP27A1 protein expression did not change significantly in DFe group, and the difference was not statistically significant; compared with HFe group, CYP27A1 protein expression was significantly lower (*P* < 0.05); CYP27B1 protein expression was highest in DFe Group, which was statistically significant compared with C, MFe and HFe groups (*P* < 0.05); compared with C group, CYP24A1 protein expression was significantly down-regulated (*P* < 0.05), CYP24A1 protein expression in MFe and HFe groups were higher than the DFe group (*P* < 0.05) (Table 6, Fig. 4).

**Table 6:** The Immunofluorescence IOD values ($\overline{x}$ ± s, n = 3)

Groups	CYP2R1	CYP27A1	CYP27B1	CYP24A1
C	207.43 ± 13.34	221.11 ± 32.88	76.76 ± 2.68	172.19 ± 12.81
DFe	59.28 ± 5.92^a^	128.94 ± 6.19	141.34 ± 5.40^a^	46.24 ± 7.75^a^
LFe	101.04 ± 5.81^ab^	192.43 ± 21.20	110.56 ± 9.71	88.59 ± 18.54^a^
MFe	278.9 ± 20.79^bc^	208.27 ± 37.14	55.24 ± 3.71^abc^	167.93 ± 27.06^b^
HFe	323.7 ± 16.37^abc^	279.64 ± 7.21^b^	33.52 ± 2.11^abcd^	191.43 ± 8.48^bc^

^a^*P* < 0.05 vs C group

^b^*P* < 0.05 vs DFe group

^c^*P* < 0.05 vs LFe group

### The mRNA expressions of CYP2R1, CYP27A1, CYP27B1, CYP24A1 were detected by q-PCR

Compared with C group, the expression of CYP2R1mRNA was down- regulated in DFe group, but the difference was not statistically significant; the content of CYP2R1mRNA in DFe and LFe groups were significantly lower than that in HFe group (*P* < 0.05); the content of CYP27A1mRNA was the lowest in DFe group, which in DFe and LFe groups were significantly lower than that in C group (*P* < 0.05); the content of CYP27B1mRNA was the highest in DFe group, and the content of CYP24A1mRNA was the lowest in DFe group, there was no statistically significant difference between the groups (Table 7).

**Table 7:** The mRNA expressions of CYP2R1, CYP27A1, CYP27B1, CYP24A1 ($\overline{x}$ ± s, n = 4)

Groups	CYP2R1	CYP27A1	CYP27B1	CYP24A1
C	1 ± 0.19	1 ± 0.09	1 ± 0.72	1 ± 0.10
DFe	0.44 ± 0.65	0.17 ± 0.01^a^	3.54 ± 1.42	0.59 ± 0.030
LFe	0.61 ± 0.07	0.32 ± 0.18^a^	2.25 ± 0.70	0.71 ± 0.260
MFe	0.96 ± 0.18	0.56 ± 0.16	1.26 ± 0.53	0.76 ± 0.110
HFe	1.80 ± 0.17^bc^	0.69 ± 0.31	1.15 ± 0.54	1.10 ± 0.180

^a^*P* < 0.05 vs C group

^b^*P* < 0.05 vs DFe group

^c^*P* < 0.05 vs LFe group

## Discussion

Iron is an essential trace element that is required for the growth and survival of the body, and plays an important role in the whole life process of the body. VD is mainly synthesized by ultraviolet rays, and environmental factors affect the seasonal fluctuation of VD concentration. The most classic role of VD is to regulate calcium and phosphorus metabolism and promote bone development. As research progresses, iron homeostasis also has a potential impact on bone formation, bone resorption and bone development [19]. The serum 1,25-(OH)_2_D_3_ concentration reduced after treatment the dietary iron deficiency in rat, based on the effect of iron deficiency on bone metabolism [20]. Iron deficiency interferes with the transformation of VD in skin and intestine and metabolism in the body [21]. Our study found that iron deficiency may affect the metabolic level of vitamin D_3_. It is suggested that iron may play a role in 25 hydroxylation by regulating CYP2R1 and CYP27A1 enzymes, and 1α-hydroxylation by regulating CYP27B1 and CYP 24A1 enzymes, thus affecting the levels of 25-(OH)D_3_ and 1,25-(OH)_2_D_3_.

Heldenberg’s [22] study on infants and young children with IDA, although supplemented with adequate VD_3_, the serum 25-(OH)D_3_ concentration of the children was still very low, and the serum 25-(OH)D_3_ concentration returned to normal after iron supplementation. In our experiment, iron deficiency led to the iron content of liver and kidney of SD rats decreased. The levels of serum 25-(OH)D_3_ and 1,25-(OH)_2_D_3_ in DFe group were significantly lower than those in C group. The levels of serum 25-(OH)D_3_ and 1,25-(OH)_2_D_3_ gradually increased with the increase of iron intervention concentration. It is suggested that iron may promote the metabolic level of VD_3_ and convert it into an active form of 1,25-(OH)_2_D_3_ in the body. In this process, iron is used by the body and the level of VD_3_ activity increases. Other studies have shown that iron loading also affects the levels of vitamin D_3_ metabolism. Napoli et al. [23] found that adults suffering from thalassemia had lower serum 25-(OH)D_3_ levels. Otto-Duessel et al. [18] observed that the lack of VD in iron overload disease was related to the accumulation of liver iron. Excessive iron accumulation in the kidney would affect VDR, which would aggravate iron deposition and cause damage to the proximal tubules of the kidney [24]. In our study, the serum 25-(OH)D_3_ and 1,25-(OH)_2_D_3_ levels in the HFe group were not found to be inhibited. It possibly because of the IDA model built in the early stage that the iron consumption in the rat body took too long. Iron only satisfied the needs of pre-consumption and growth and development requirements, and could not accumulate too much in the body, and thus did not inhibit the serum 25-(OH)D_3_ and 1,25-(OH)_2_D_3_ levels.

Our study found that iron deficiency reduced the iron concentration in the liver and kidney, the SI concentration was increased accordingly, the SI concentration in HFe group is lower than that in LFe group and MFe group, which is consistent with the study obtained by DiJiong et al. [25]. In this study, the down-regulation of the expression of CYP2R1 and CYP27A1 at the protein and gene levels, the up-regulation of the expression of CYP27B1 and the expression of VD catabolic enzyme CYP24A1 was down-regulated accordingly. This is because VD has hormonal properties and there is feedback regulation in the body. In order to maintain the stability of hormone level in the body, when 1,25-(OH)_2_D_3_ caused by iron deficiency is low to a certain value, it will cause the result of feedback regulation. This phenomenon suggested that under the condition of sufficient iron, the hydroxylation of VD is the use of this metal enzyme, which may effectively carry out hydroxylation reaction; when iron deficiency, hydroxylase may not rely on iron.

In this study, serum 25-(OH)D_3_ and 1,25-(OH)_2_D_3_ levels were significantly correlated with TIBC, TF and TFR, but such correlation was not found with SI, indicating that there was a some correlation between vitamin D_3_ metabolism and iron. We think that reasonable iron supplementation to increase the level of VD activity is a simple, safe, cheap and effective method. At the same time, accurate identification of VD hydroxylation cofactors improves the bioavailability of VD, and the participation of iron in the formation of CYP450 enzymes plays a key role in VD metabolism. In our study, iron-deficient feed was used to establish an IDA model, the method reduced the levels of SI and Hb in rats at a stable rate, with no trauma and reliable effects.

Our study takes the lead in investigate the systematic mechanism of iron on VD metabolism. However, our research also had certain limitations, we only based on animal experiments, the digestibility and absorptivity of iron and VD metabolism in group C and group M were not studied. It is necessary to further explore the effect of iron on VD metabolism in the population, so that proper iron supplementation can promote the expression of VD active products in the VD deficiency patient population. To understand the potential benefits of VD deficiency, provide new ideas for VD deficiency diseases.

## Conclusions

Iron deficiency reduces iron reserves in liver and kidney, which may affect hydroxylase activity; Iron may reduce the levels of 25-(OH) D_3_ and 1,25-(OH)_2_D_3_ by regulating hydroxylase at the translation and transcription levels; It is illustrated that the decrease of iron reserve may be a necessary condition for the control of these hydroxylase enzymes, even in the case of mild iron deficiency, the enzyme function may change.

## Supplementary Information

**Additional file 1:** Weight changes before and after intervention.

## Data Availability

The datasets used during the present study are available from the corresponding author upon reasonable request.

## Abbreviations

VD: Vitamin D
Hb: Hemoglobin
BRC: Red blood cell
SI: Serum iron
TIBC: Iron binding capacity
TF: Transferrin
Tfr: Transferrin receptor
IDA: Iron deficiency anemia
SPF: Specific pathogen free
RIPA: Immunoprecipitation Assay
DAPI: Diamidino-2-pheny1 indole
CYP450: Cytochrome P450
IOD: Integrated optical density
C: Control
DFe: Deficiency iron
LFe: Low iron
MFe: Medium iron
HFe: High iron
