# Supplementary material for: The effect of iron dextran on vitamin D3 metabolism in SD rats
Source: Nutr Metab (Lond). 2022 Jul 16;19:47. doi: 10.1186/s12986-022-00681-5 (PMC9288701; doi:10.1186/s12986-022-00681-5)
Supplement: Supplementary file 1 — Additional file 1: Weight changes before and after intervention. [file 12986_2022_681_MOESM1_ESM.docx]

Weight changes before and after intervention

Table 1 Body weight changes in 1-6 weeks

| Groups | 1w | 2w | 3w | 4w | 5w | 6w |
| --- | --- | --- | --- | --- | --- | --- |
| C | 89.99±4.83 | 136.74±10.60 | 176.65±16.42 | 219.18±18.64 | 270.06±22.51 | 313.85±22.82 |
| M | 89.33±5.41 | 137.45±10.69 | 164.61±12.91 | 198.92±18.55^*^ | 244.20±19.96^*^ | 273.31±24.96^*^ |

^*^*P＜*0.05 vs C group

Table 2 Body weight changes in 7-10 weeks

| Groups | 7w | 8w | 9w | 10w |
| --- | --- | --- | --- | --- |
| C | 339.24±18.63 | 369.42±21.50 | 373.57±20.70 | 376.91±13.59 |
| DFe | 303.19±30.01^*^ | 326.71±30.91^*^ | 332.54±24.67^*^ | 339.15±22.54^*^ |
| LFe | 301.58±28.23^*^ | 327.87±33.50^*^ | 340.74±34.83^*^ | 344.11±31.34^*^ |
| MFe | 306.97±18.48^*^ | 339.23±21.04^*^ | 348.76±23.54 | 350.91±24.62 |
| HFe | 305.77±27.36^*^ | 346.32±34.82 | 350.91±47.50 | 355.53±47.21 |

^*^*P＜*0.05 vs C group
